# Clinical educators’ perspectives on physical activity and exercise prescription in clinical education for physiotherapy: A qualitative study

**DOI:** 10.4102/sajp.v82i1.2272

**Published:** 2026-03-27

**Authors:** Shamila Gamiet, Farhana Karachi, Joliana Phillips, José Frantz

**Affiliations:** 1 Interprofessional Education Unit, Faculty of Community and Health Sciences, University of the Western Cape, Bellville, South Africa; 2 Department of Physiotherapy, Faculty of Community and Health Sciences, University of the Western Cape, Bellville, South Africa; 3 Research Development and Postgraduate Support Office, University of the Western Cape, Bellville, South Africa; 4 Office of the Deputy Vice-Chancellor of Research and Innovation, University of the Western Cape, Bellville, South Africa

**Keywords:** clinical educators, exercise prescription, health promotion, physical activity, physiotherapy clinical education, non-communicable diseases

## Abstract

**Background:**

Physical activity (PA) promotion and exercise prescription (EP) are crucial core competencies in addressing the growing burden of non-communicable diseases. However, the extent to which these competencies are taught and implemented in clinical education for physiotherapy remains unclear within the South African context.

**Objectives:**

To explore clinical educators’ perceptions regarding PA and EP in clinical education for physiotherapy at a local university in the Western Cape, South Africa.

**Method:**

A qualitative exploratory descriptive design was employed. Eleven clinical educators participated in two focus group discussions.

**Results:**

Participants had an average of 15 years of clinical experience. More women (73%, *n* = 8/11) participated. Thematic analysis revealed three principal themes: (1) Professional identity and patient empowerment, (2) knowledge translation challenges: bridging theory and practice and (3) time and resource constraints – identifying practical constraints that impact teaching and implementation. Participants expressed their profession’s responsibility in promoting PA and prescribing exercises across all clinical settings. They identified challenges in clinical practice, knowledge translation gaps and the need for strengthened professional development.

**Conclusion:**

The complex interplay between educational theory, clinical practice constraints and the evolving role of physiotherapists in public health are highlighted. A multi-faceted approach that considers educational reform and healthcare system constraints, while maintaining a focus on improving patient outcomes through promoting PA and exercise, is recommended.

**Clinical implications:**

Emphasis on preparing physiotherapy students to effectively educate and empower patients for independent health management is recommended and could impact population health outcomes in resource-limited settings in which the ongoing professional supervision of exercise programmes is often not feasible.

## Introduction

Non-communicable diseases (NCDs) represent a growing public health crisis globally, with physical inactivity identified as a leading risk factor for NCD mortality (DiPietro et al. 2020; Katzmarzyk et al. 2022; World Health Organization 2020). Regular exercise and an active lifestyle can significantly reduce the risk of NCDs, such as obesity, diabetes, cardiovascular disease, cancers and mental illness (Jung et al. 2024; Pedersen & Saltin 2015; Rooney, Gilmartin & Heron 2023). Despite the World Health Organization’s (WHO) recommendation that adults engage in at least 150–300 min of moderate-intensity aerobic exercise throughout the week for substantial health benefits, 28% of adults globally remain physically inactive, with no improvement since 2001 (Bull et al. 2020; Guthold et al. 2018; WHO 2020). Persistent sedentary lifestyles threaten public health outcomes and necessitate urgent intervention strategies, particularly as the WHO has set targets to reduce physical inactivity levels by 15% in adults and adolescents by 2030 (WHO 2024).

Physical activity (PA) promotion and exercise prescription (EP) are recognised as core competencies in physiotherapy education and practice, representing well-established strategies in health promotion and disease prevention (Corey et al. 2022; Lowe, Littlewood & McLean 2018; McTiernan et al. 2019; Rooney et al. 2023). The physiotherapy profession must expand its scope from traditional tertiary prevention – managing and rehabilitating existing conditions – to encompass primary prevention and health promotion (Corey et al. 2022; Holm et al. 2015). This evolution positions physiotherapists as key contributors to addressing the NCD burden through evidence-based PA interventions. The World Confederation of Physical Therapy (WCPT) recognises physiotherapists’ integral role in promoting PA and EP, recommending that these competencies be comprehensively covered in physiotherapy education to prepare students for contemporary professional practice (Dean et al. 2014; O’Donoghue, Doody & Cusack 2011, 2012; WCPT 2019). Physiotherapists are uniquely positioned in hospitals and communities to support both prevention and management of NCDs through PA and EP interventions (Dean et al. 2014; Lowe et al. 2018; Shirley, Van der Ploeg & Bauman 2010; Taukobong et al. 2014; West et al. 2021).

However, significant gaps exist in translating PA and EP knowledge into effective clinical practice (Wing et al. 2025). Despite strong evidence for exercise as a therapeutic tool, it remains underutilised in clinical settings (Dean 2009; Khan, Weiler & Blair 2011; Lowe et al. 2018; Wing et al. 2025). While physiotherapy curricula include theoretical frameworks for health promotion, the extent to which PA and EP competencies are effectively taught and implemented remains unclear, particularly in resource-constrained contexts like South Africa (Bodner et al. 2013; Taukobong et al. 2014). The integration of health promotion concepts into physiotherapy education is considered essential for enhancing patient self-empowerment and independence, yet uncertainty remains about how comprehensively these strategies are embedded into academic curricula and clinical practice training (Mokwena & Phetlhe 2015). Clinical educators play a crucial role in shaping students’ knowledge, attitudes and beliefs regarding PA promotion and EP, yet their perspectives on PA and EP education remain largely unexplored (Sajjad et al. 2024).

The South African context presents unique challenges, with a rapidly rising NCD burden and constrained healthcare resources. Given South Africa’s high burden of chronic diseases of lifestyle, there is particular importance to equipping physiotherapists with practical skills to implement health promotion strategies, including PA and EP in clinical practice (Walkeden & Walker 2015). Despite robust evidence supporting PA benefits, there remains a noticeable lack of urgency in South Africa to prioritise PA promotion, evident in the limited body of local research examining health impacts within the South African context and the absence of a coordinated national strategy leveraging exercise as a preventive intervention (Patricios, Saggers & Torres 2022). Understanding clinical educators’ knowledge, attitudes and beliefs regarding PA and EP is vital for several reasons: clinical educators serve as role models during clinical training, identifying gaps can inform targeted professional development initiatives and insights can help bridge the theory–practice gap in healthcare education (Alshehri & Alzahrani 2024; Sajjad et al. 2024; Schwab et al. 2022).

Our study addresses a significant gap in physiotherapy education literature by exploring clinical educators’ perspectives on PA and EP education. While studies have explored EP practices among physiotherapists in clinical settings, there is a significant gap regarding how clinical educators perceive and teach these skills within physiotherapy clinical education programmes (Lowe et al. 2018). This gap is particularly pronounced in the South African context, in which previous research has examined students’ perspectives on PA and EP education, but the voices of clinical educators remain largely unexplored (Shirley et al. 2010). By examining clinical educators’ knowledge, attitudes and beliefs regarding PA and EP at the University of the Western Cape (UWC), this research aims to inform curriculum development and professional preparation strategies that better equip graduates to address South Africa’s NCD burden through effective PA promotion and EP implementation. Addressing clinical educators’ challenges through targeted training and curriculum updates is essential for preparing graduates to be proficient in promoting PA and prescribing exercises in clinical practice.

## Research methods and design

### Study design

A qualitative approach using an exploratory descriptive study design was employed. Focus group discussions were selected to collect data. Focus group discussions enable participants to share their perspectives and experiences and facilitate a deeper understanding of perspectives (Gill et al. 2008; Levitt et al. 2017; Mohajan 2018).

### Participant sampling and recruitment strategy

The target population included 22 (*n* = 22) clinical educators employed by the UWC Physiotherapy Department to provide clinical supervision to undergraduate students in clinical practice. Eighteen (*n* = 18) physiotherapy clinical educators were employed on a part-time basis, and four (*n* = 4) were full-time academic staff involved in the clinical supervision of undergraduate students as part of their workload. Purposive (non-probability, total inclusive) sampling was used to recruit all 22 participants, who had knowledge of clinical education and the curriculum at the included physiotherapy department. We acknowledge that while our study was limited to clinical educators from a single institution, this approach allowed for in-depth exploration of institution-specific challenges and opportunities. The participants represented diverse clinical experience levels and educational backgrounds, providing varied perspectives within the institutional context.

### Data collection procedure

The total study population of 22 clinical educators was invited via email to participate in our study. Eleven agreed to partake. Two focus groups were conducted; firstly, the session was performed in person at the Department of Physiotherapy at UWC on a day and time that was convenient for all consenting participants (*n* = 6), and secondly, it was performed virtually using Google Meet (*n* = 5). The in-person focus group was facilitated by the research supervisor J.P., while the primary author S.G. observed. The online focus group was conducted via Google Meet and facilitated by the primary author S.G. Each focus group session started with a short introduction by the facilitator to outline the key rules for the focus group. Focus groups lasted approximately 1 h and were audio recorded with the permission of all participants. A semi-structured interview guide, informed by existing literature on the topic and aligned with our study objectives, was used to generate open discussion. Pseudonyms were assigned and used throughout our study to protect participants’ identities. At the end of each focus group, participants were thanked for their contribution and informed that the data would be returned to them for review once transcribed. Data saturation was evaluated by examining when no new themes or significant variations emerged from the data. After the second focus group, the research team reviewed all themes and determined that sufficient depth and breadth had been achieved to address the research objectives. While theoretical saturation may not have been fully achieved due to the limited number of focus groups, informational saturation was reached regarding the core themes.

### Data management and analysis

**FIGURE 1:**
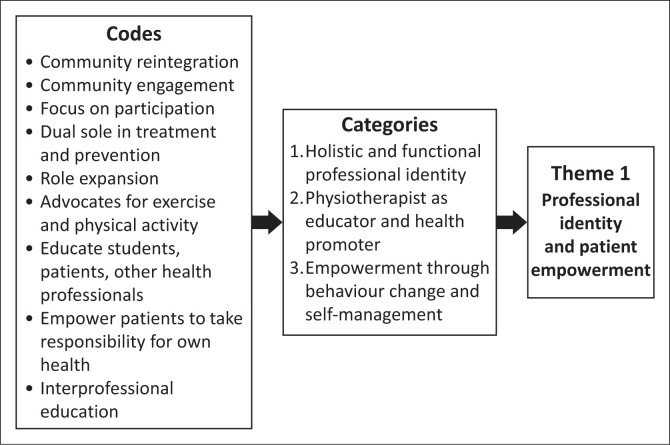
Codes and categories generating Theme 1.

**FIGURE 2:**
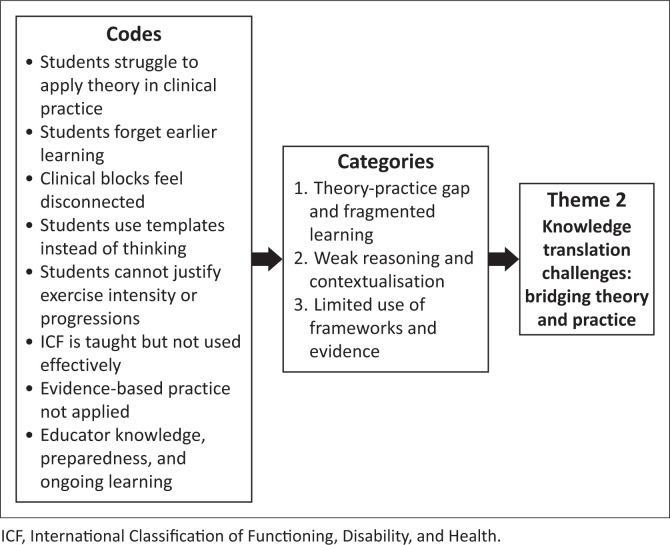
Codes and categories generating Theme 2.

**FIGURE 3:**
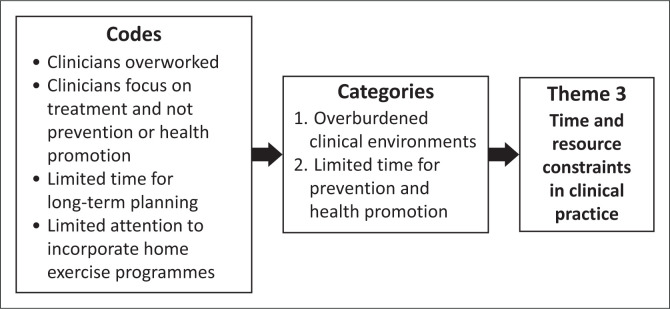
Codes and categories generating Theme 3.

The audiotaped recordings from the two focus groups were transcribed verbatim by an independent transcriber. The audio recordings were cross-checked against the transcripts by S.G. to ensure accuracy. Participants were then given the opportunity to review the authors’ interpretations of the data to verify accuracy. S.G. read through the transcripts multiple times to become familiar with the data. Manual thematic analysis was conducted by S.G., using Braun and Clarke’s (2006) six-step method employing an inductive approach that enabled patterns to emerge directly from the data (Braun & Clarke 2019). Braun and Clarke (2021) emphasise the natural, organic development of themes throughout the inductive, analytical process. Inductive coding was guided by the research aims. The coded transcripts were reviewed by J.P., who was familiar with the topic and had experience in qualitative research. This review served as a form of peer debriefing that offered alternative perspectives on the data and encouraged reflexivity (Braun & Clarke 2019; McMahon & Winch 2018). The codes were then actively developed into broader themes through a process of interpretation and refinement, after which they were reviewed again by J.P. These codes, their categories and the final themes generated are described in Figure 1, Figure 2 and Figure 3.

Throughout the data collection and analysis, S.G. engaged in ongoing reflexivity to ensure that personal interest in PA and EP in clinical education did not unduly influence the process or findings. This aim was facilitated through the use of a reflexive diary, which was regularly discussed with researcher supervisor J.P., to maintain an appropriate level of detachment at critical stages. The trustworthiness of our study was enhanced using Guba’s framework, which includes credibility, transferability, dependability and confirmability. Credibility was supported by participants’ verification of accurate data interpretations. Transferability was supported by providing detailed descriptions of the research context, sampling and analytical process, allowing for assessment of relevance to other contexts. Dependability was achieved through comprehensive documentation of the research process, ensuring transparency and reproducibility. Confirmability was strengthened by maintaining an audit trail that captured the progression of data analysis and interpretive reasoning. The integration of verbatim participant quotations and transparent methodological reporting further reinforced our study’s integrity.

### Ethical considerations

The research was approved by the Humanities and Social Sciences Research Ethics Committee of the UWC (Ethics Clearance Number: HS18/8/10). Participants received information sheets outlining our study’s purpose and procedures. Written informed consent was obtained in hard copy or digitally from all participants. Transcripts were anonymised and stored securely in a password-protected folder on Google Drive to protect confidentiality. Participants were informed that their participation was entirely voluntary, with no impact on their academic status or professional relationships, and that they could withdraw at any time without consequence.

## Results

Eleven clinical educators participated in two focus group discussions – six in the first and five in the second. Our study sample reflected a strong representation of clinical expertise across specialities, including neuromusculoskeletal physiotherapy, paediatrics, neurology, orthopaedics, cardio-pulmonary rehabilitation and critical care. Most participants were women (73%, *n* = 8), with men comprising 27% (*n* = 3). The group had an average of 15 years of clinical experience. In terms of qualifications, 18% held doctoral degrees, 18% held master’s degrees and 64% held bachelor’s degrees with honours in physiotherapy. Table 1 summarises the socio-demographic characteristics of the participants.

**TABLE 1:** Participants’ socio-demographic information (*n* = 11).

No	Gender	Educational background	Years of clinical work experience	Clinical expertise
1	Female	BSc Physiotherapy	15	Neuromusculoskeletal
2	Female	PhD Physiotherapy	13	Neuromusculoskeletal
3	Female	BSc Physiotherapy	15	Cardio-pulmonary rehabilitation Critical care (acute spinal cord injuries)
4	Male	MSc Physiotherapy	6	Paediatrics, Sports Rehabilitation
5	Male	MSc Physiotherapy	9	Exercise Physiology, Cardio-pulmonary rehabilitation
6	Male	PhD Physiotherapy	27	Neurology
7	Female	BSc Physiotherapy	32	Paediatrics
8	Female	BSc Physiotherapy	8	Paediatrics
9	Female	BSc Physiotherapy	9	Orthopaedics, Neurology, Cardio-pulmonary rehabilitation, Neuromusculoskeletal
10	Female	BSc Physiotherapy	17	Cardio-pulmonary rehabilitation Critical Care (medical, surgical, neurology and cardiothoracic)
11	Female	BSc Physiotherapy	12	Orthopaedics, Neurology, Cardio-pulmonary rehabilitation, Paediatrics

BSc, Bachelor of Science; PhD, Doctor of Philosophy; MSc, Master of Science.

Thematic analysis of the data revealed three principal themes that capture clinical educators’ perspectives on PA and EP in physiotherapy clinical education: (1) Professional identity and patient empowerment, (2) knowledge translation challenges: bridging theory and practice and (3) time and resource constraints – identifying practical constraints that impact teaching and implementation. These themes collectively illustrate the complex interplay between educational theory, clinical practice realities and the evolving professional identity of physiotherapists in health promotion. Each theme is presented below with supporting evidence from participant voices.

### Theme 1: Professional identity and patient empowerment

Participants emphasised the evolving professional identity of physiotherapists as advocates for PA promotion and EP, extending beyond traditional rehabilitation to encompass primary prevention, public health leadership and patient empowerment (Figure 1). This expanded role requires physiotherapists to not only prescribe exercises but also foster patient self-efficacy and responsibility for long-term health management. Clinical educators recognised that while PA promotion and EP are core physiotherapy competencies, realising this professional identity requires both advocacy within the healthcare system and effective patient education strategies.

Participants articulated their profession’s essential role in prescribing exercise across diverse clinical contexts:

‘I think as physiotherapists, we do have an exceptionally important role to play in prescribing exercises. Also, if you look at the setting in which you work; for example, if you’re, maybe, in an acute setting, or if you’re working within a multi-disciplinary team, just for patient wellness, whether it’s maintenance, endurance, or something more serious as a spinal cord injury then that becomes our key focus, actually.’ (Participant 3, focus group 1, female, 15 years clinical experience)

Beyond individual patient care, participants advocated for a paradigm shift towards preventive care and public health approaches, positioning physiotherapists as leaders in community health promotion:

‘Just prevention at a primary level, as opposed to, I think it’s already too late when you start seeing the patient when they’re in hospital and they have been diagnosed with diabetes, or any other chronic lifestyle disease. So, I think our role should be more geared to public health, being a public health specialist, as opposed to just treating patients.’ (Participant 5, focus group 1, male, 9 years clinical experience)

Central to this expanded professional identity is the responsibility to educate multiple stakeholders about the importance and benefits of PA promotion and EP, extending advocacy across professional boundaries:

‘I think it’s not just educating the patient as such, but also educating our fellow colleagues, like the doctors or physicians. A lot of them don’t know that physical activity is an aspect that physiotherapy can dive into, which will actually benefit and help the patient later on in life.’ (Participant 3, focus group 1, female, 15 years clinical experience)

‘I think, as we said, we really need to educate our students about the benefits of physical activities. I have an impression that some of them don’t know what it is, I mean, what are the benefits. So, if they don’t know what are the benefits of physical activity, then it would be very difficult for them to educate the patients.’ (Participant 6, focus group 1, male, 27 years clinical experience)

Participants recognised that effective EP must ultimately empower patients to take ownership of their health management, particularly given resource constraints in healthcare settings:

‘It is important to prescribe exercises because I found that it gives the patient a sense of responsibility for their own health. I think patients can now take responsibility for themselves once you’ve given them an effective home exercise programme.’ (Participant 9, focus group 2, female, 9 years clinical experience)

This emphasis on patient empowerment reflects the understanding that sustainable health outcomes require patients to become active participants in their care, moving beyond passive treatment recipients to informed self-managers of their PA and exercise routines.

### Theme 2: Knowledge translation challenges: Bridging theory and practice

Participants identified challenges in translating theoretical knowledge about PA and EP into clinical practice (Figure 2). While acknowledging that the undergraduate curriculum provides adequate content on EP and theoretical frameworks such as the International Classification of Functioning, Disability, and Health (ICF), both students and clinical educators struggle to apply this knowledge comprehensively in clinical settings. This theme reveals a persistent theory–practice gap that affects both the learning and the teaching of PA promotion and EP competencies.

Clinical educators noted that students often compartmentalise their learning, failing to integrate knowledge across different modules and clinical contexts:

‘Exercise Physiology speaks about the World Health Organisation recommendations of doing 150 minutes of physical activity per week. We also try to incorporate some of the latest evidence between exercise prescriptions in different special population groups, but sometimes the students fail to translate that to the other setting, like the clinical practice.’ (Participant 5, focus group 1, male, 9 years clinical experience)

This compartmentalisation extends beyond individual modules, with students struggling to carry forward foundational concepts throughout their academic progression:

‘They don’t make the effort to bring everything they’ve learnt with them every year, every step of the way. They leave this module there in the first year in the first term, and then when they get to third year and fourth year and have to apply it, and then it’s like: oh, we did do that, I think, it sounds familiar.’ (Participant 1, focus group 1, female, 15 years clinical experience)

While theoretical frameworks like the ICF are taught to guide holistic patient management, participants observed that both students and practitioners often focus narrowly on impairments while neglecting broader functional improvements through EP:

‘We focus so much on the ICF, and we use the ICF as a framework to treat our patients. Or we should be using the ICF to treat our patients. We want to re-integrate them back into their communities, and into their previous roles, then exercise prescription becomes important. We basically are good at assisting at the impairment level hands-on, but after that, this patient needs to take some responsibility, or needs to have something to do that can help them if we’re not there or we can’t assist them. So, for me then, exercise prescription and knowledge of exercise prescription becomes important for the practitioner.’ (Participant 2, focus group 1, female, 13 years clinical experience)

Significantly, clinical educators acknowledged their own knowledge gaps in EP, highlighting that the theory–practice challenge extends beyond student learning to educator preparedness:

‘I wasn’t really taught types of exercises, so I relied on Google after I graduated. It wasn’t really something that was covered during my undergrad, so I do think there’s a need for exercise prescription to be taught to the students, undergrad. I think if there are, like, little workshops and stuff that could potentially be done, specifically on exercise prescription or types of exercise. You know, I rely on my second year, which is almost twenty years ago, knowledge, you know, things could have changed by now.’ (Participant 10, focus group 2, female, 17 years clinical experience)

To address these challenges, participants suggested that health promotion and EP concepts should be integrated throughout the curriculum rather than confined to specific modules:

‘It’s almost in every module that is being taught, from first year right through to fourth year, that health promotion needs to be included in every module. There’s a Health Promotion module in second year that’s very specific, and it doesn’t get carried through, it gets left behind in second year, and nobody’s taking it forward in other modules in third and fourth year.’ (Participant 4, focus group 1, male, 6 years clinical experience)

### Theme 3: Time and resource constraints in clinical practice

Time constraints emerged as a significant challenge, with participants describing the difficulties of incorporating a comprehensive EP into busy clinical practices (Figure 3). In acute care settings, the pressure to facilitate early patient discharge often results in limited time for developing long-term exercise programmes:

‘At a day hospital, the setting is a rushed setting, so there are queues of people waiting to be seen, and there’s frustration because the students do take longer than the clinician at the setting would. So, that is already putting pressure on the students, so all they want to do is get through what is happening with the patients. Like, for example, pain levels, what they can do now for the patient, where exercise then becomes a long-term treatment.’ (Participant 1, focus group 1, female, 15 years clinical experience)

‘I also think that clinicians in hospital settings are so overworked that they might not find the time or feel that this is important, because they are treating patients to get them out of hospital. They’re not necessarily looking at prevention. Clinicians don’t always have that time to take it that one step further.’ (Participant 4, focus group 1, male, 6 years clinical experience)

The focus on immediate management often overshadows long-term exercise planning:

‘So, you focus on managing the patient at that moment. I spend quite a lot of time chatting through the problem list, and the long-term and short-term goals. And when we discuss long-term goals, we speak a lot about home exercise programmes. And where they see the patient in two, three, four, five months’ time, but it’s difficult for the student sometimes to envision where the patient is going to end up. But it doesn’t play as big a part as the current management of the patient.’ (Participant 9, focus group 2, female, 9 years clinical experience)

## Discussion

Our study provides valuable insights into clinical educators’ perspectives on PA promotion and EP in physiotherapy education within the South African context. The three themes that emerged reveal interconnected challenges that collectively impact the effectiveness of PA and EP education: evolving professional identity expectations, persistent knowledge translation barriers and systemic resource constraints. These findings contribute significantly to understanding how contextual factors influence clinical education delivery in resource-limited settings.

### Professional identity evolution and educational implications

The emphasis on professional identity and patient empowerment reflects a global shift in physiotherapy practice towards primary prevention and public health advocacy (Dean 2009; WCPT 2019). Our findings align with recent literature, recognising physiotherapists as experts in PA promotion and EP across healthcare settings (Barton et al. 2021; Shirley et al. 2010; West et al. 2021). Furthermore, physiotherapists, as movement specialists, are well placed to combat health risks related to sedentary lifestyles (Lowe et al. 2018; Shirley et al. 2010; West et al. 2021). This shift is timely, given the WHO’s recognition of physical inactivity as a major contributor to NCD mortality worldwide (WHO 2024). Integrating patient empowerment into the physiotherapy role reflects a more holistic view of healthcare – one that moves beyond traditional biomedical models. Participants in our study highlighted that prescribing exercise effectively is not just about clinical skills but also about supporting behaviour change and facilitating the self-efficacy of patients. This attitude reflects a patient-centred approach that acknowledges individuals as active partners in their health management. This view aligns with comprehensive evidence supporting exercise as medicine and emphasising the importance of patient engagement in achieving sustainable health outcomes (Al-Ghafri et al. 2021; Pedersen & Saltin 2015; Varkey et al. 2022). The shift towards patient empowerment is especially important in resource-constrained environments where patients may not have access to physiotherapy services and may need to take responsibility for their own health (Chong et al. 2020; Mokwena & Phetlhe 2015). Therefore, rethinking traditional physiotherapy curricula is crucial. The inclusion of health promotion, patient education and behaviour change strategies in physiotherapy education is imperative to prepare graduates to function as health advocates and leaders in promoting PA and prescribing exercise and is supported by Walkeden and Walker (2015).

### Knowledge translation challenges in resource-constrained settings

The persistent theory–practice gap identified in our study reflects broader challenges documented internationally in health professional education (O’Donoghue et al. 2011; Schwab et al. 2022). However, our findings extend this understanding by revealing how resource constraints in South African healthcare settings compound these challenges. The compartmentalisation of learning observed among students, in which knowledge from early modules fails to integrate into clinical practice, suggests fundamental issues with curriculum design and delivery. Particularly concerning is the acknowledgement by clinical educators of their own knowledge gaps in prescribing exercise. This finding is supported by Barton et al. (2021), who surveyed physiotherapists across 56 countries and found inconsistencies in knowledge, competence and application of exercise guidelines. Similarly, an online survey by Chesterton, Alexanders and Alexanders (2023) found that United Kingdom physiotherapy graduates felt underprepared in EP. Collectively, these studies reveal systemic educational gaps in EP across contexts. In the South African context, where healthcare resources are constrained and the burden of lifestyle-related NCDs is rising rapidly (Patricios et al. 2022), such knowledge gaps among educators create a cascade effect that ultimately impacts patient care quality.

The challenge extends beyond individual knowledge deficits to systemic issues with framework application. While participants acknowledged the importance of the ICF framework for holistic patient management, they noted a persistent focus on impairment-level interventions at the expense of participation and community reintegration goals. This disconnect between theoretical frameworks and practical application reflects what Narain and Mathye (2023) describe as the need for transformation in South African physiotherapy education to better align with primary healthcare principles. These principles emphasise equity, accessibility, community participation and a focus on prevention and health promotion – core values underpinning person-centred, community-oriented care. Aligning physiotherapy education with primary healthcare principles requires curricula and clinical training that integrate prevention, participation and social determinants of health to prepare physiotherapists for practice in resource-limited settings.

### Systemic barriers and healthcare context

The time and resource constraints identified in our study reflect broader challenges in South African healthcare delivery, which extend beyond educational settings. The pressure for rapid patient turnover in acute care settings, combined with limited staffing and resources, creates environments where comprehensive EP becomes secondary to immediate symptom management. This finding resonates with international literature identifying similar barriers to PA promotion in clinical practice (Alshehri & Alzahrani 2024; Lowe et al. 2018). These barriers align with challenges identified in other African contexts, in which physiotherapists report similar time constraints and training needs for effective PA promotion (Abaraogu, Edeonuh & Frantz 2016). The systemic nature of these constraints suggests that they are embedded within healthcare financing models that prioritise volume over quality, creating perverse incentives that undermine comprehensive patient care approaches.

However, our study reveals how these systemic barriers specifically impact clinical education delivery, creating what could be termed an ‘educational poverty cycle.’ Students learning in high-pressure environments with limited time for comprehensive patient assessment and long-term planning are unlikely to develop the skills and confidence needed for effective PA promotion and EP in their future practice. This educational deficit is compounded by the clinical educators, who themselves lack adequate time to model best practices or provide detailed mentorship on EP techniques. The result is a concerning cycle in which systemic constraints perpetuate the inadequate preparation of future practitioners, who then enter the workforce unprepared to challenge or change existing practice patterns, thereby reinforcing the status quo of suboptimal PA and exercise implementation.

### Integrated implications for physiotherapy education

The interconnected nature of our themes suggests that addressing challenges in PA and EP education requires comprehensive, multi-level interventions rather than isolated curricular changes. Developing professional identity depends on addressing knowledge gaps, as confidence and competence in EP underpin physiotherapists’ role as health promoters. Without current evidence-based knowledge of EP, clinical educators and students cannot fully realise the preventative and advocacy aspects of physiotherapy practice. Ongoing professional learning and curriculum renewal are therefore essential to strengthening the profession’s evolving identity. Effective knowledge translation, likewise, depends on supportive clinical learning environments that provide students with opportunities to apply theoretical principles of PA promotion and EP under guided supervision. When clinical educators model best practice, foster interdisciplinary collaboration and prioritise reflection and feedback, theoretical knowledge is effectively translated into clinical competence. In their absence, theory remains disconnected from practice. Collectively, these findings point to a systemic need for transformation in health professional education – one that aligns with broader calls that integrate social determinants of health and contextual realities of healthcare delivery (Schwab et al. 2022). Our findings support recommendations for the longitudinal integration of PA and EP concepts throughout physiotherapy curricula rather than compartmentalised modules (O’Donoghue et al. 2011). This integration must extend beyond theoretical knowledge to include practical application opportunities, behaviour change strategies and public health perspectives.

The knowledge gaps identified among clinical educators highlight an urgent need for targeted professional development programmes. Such initiatives should address both technical EP knowledge and pedagogical approaches for teaching these skills in resource-constrained environments. These programmes must incorporate evidence-based EP principles, contemporary behaviour change strategies and innovative teaching methodologies that maximise learning within time-limited clinical placements while ensuring that educators can confidently model and teach comprehensive PA promotion skills.

Addressing systemic barriers requires collaboration between educational institutions and clinical placement sites to develop innovative approaches for teaching comprehensive PA and exercise within existing time and resource constraints. This teaching might include structured protocols for EP that acknowledge clinical realities while maintaining educational objectives. Potential solutions could involve developing standardised assessment templates that efficiently capture PA promotion opportunities, creating digital resources for rapid EP reference and establishing dedicated time slots for PA-focused patient interactions. Collaborative initiatives might also include joint training programmes for clinical educators, shared curriculum development that integrates workplace learning with academic theory and the establishment of community-based learning sites where students can practise PA promotion in less time-pressured environments. Furthermore, partnerships could facilitate the development of technology-enhanced learning tools, such as mobile applications for EP or virtual reality simulations that allow students to practise patient education scenarios. These collaborative efforts should aim to create sustainable models that benefit both educational quality and clinical service delivery, ultimately producing graduates better prepared for contemporary physiotherapy practice while improving patient care outcomes in resource-constrained settings.

### Implications for the South African context

Our findings have particular relevance for South African physiotherapy education, given the country’s unique healthcare challenges. The high burden of lifestyle-related NCDs, combined with resource constraints and healthcare disparities, creates both an urgent need and significant barriers for effective PA and exercise implementation. The evolution of physiotherapists’ professional identity towards public health advocacy may be particularly important in the South African context, where physiotherapists are often among the few healthcare professionals accessible to communities. The emphasis on patient empowerment and self-efficacy identified in our study may be especially relevant in resource-limited settings in which the ongoing professional supervision of exercise programmes is often not feasible. Preparing physiotherapy students to effectively educate and empower patients for independent health management could significantly impact population health outcomes.

### Study limitations and future directions

While our single-institution study provides valuable insights into institution-specific factors influencing PA promotion and EP education, the findings’ generalisability is limited. Future multi-site studies across diverse South African physiotherapy programmes would enhance the understanding of how different institutional and regional contexts influence clinical education delivery. In addition, while data saturation principles guided our analysis, we acknowledge that additional focus groups may have provided further nuanced insights. However, the consistency of themes across both groups and the depth of discussion achieved suggest adequate informational saturation for our exploratory study.

The theory–practice gap identified in our study warrants investigation of specific educational interventions. Future research should explore innovative pedagogical approaches for enhancing knowledge translation and evaluate their effectiveness in improving students’ PA and EP competencies. Additionally, longitudinal studies tracking graduates’ actual exercise promotion and prescription practices would provide valuable insights into the long-term impact of educational interventions.

## Conclusion

Our study reveals the complex interplay between professional identity evolution, knowledge translation challenges and systemic constraints in shaping PA and EP education within South African physiotherapy programmes. The findings suggest that effective educational reform requires comprehensive approaches addressing individual, institutional and systemic factors. As physiotherapy continues evolving towards greater emphasis on health promotion and primary prevention, educational programmes must adapt to prepare practitioners who can effectively advocate for and implement PA and EP interventions despite resource constraints. These efforts are essential for positioning physiotherapists as effective contributors in addressing South Africa’s growing burden of lifestyle-related NCDs.
